# BM-MSC-derived small extracellular vesicles (sEV) from trained animals presented nephroprotective potential in unilateralureteral obstruction model

**DOI:** 10.1590/1678-9199-JVATITD-2020-0187

**Published:** 2021-12-03

**Authors:** Rafael da Silva Luiz, Rodolfo Rosseto Rampaso, Alef Aragão Carneiro dos Santos, Marcia Bastos Convento, Dulce Aparecida Barbosa, Cassiane Dezoti da Fonseca, Andréia Silva de Oliveira, Agnaldo Caires, Andrei Furlan, Nestor Schor, Fernanda Teixeira Borges

**Affiliations:** 1 Nephrology Division, Department of Medicine, Federal University of São Paulo (UNIFESP), São Paulo, SP, Brazil.; 2 Interdisciplinary Program in Health Sciences, Institute of Physical Activity and Sport Sciences, Cruzeiro do Sul University, São Paulo, SP, Brazil.; 3 Paulista School of Nursing, Federal University of São Paulo (UNIFESP), São Paulo, SP, Brazil.

**Keywords:** Chronic kidney disease, Physical activity, Bone marrow mesenchymal stromal cells, Small extracellular vesicles, Angiogenesis

## Abstract

**Background:**

The efficacy of bone marrow mesenchymal stromal cells (BM-MSC) and its extracellular vesicles has been demonstrated for a broad spectrum of indications, including kidney diseases. However, BM-MSC donor characteristics and their potential are not usually considered. Therefore, the present work aims to evaluate the nephroprotective capacity of sEV secreted by BM-MSC from trained rats inunilateral ureteral obstruction (UUO) model.

**Methods:**

BM-MSC was characterized by their differentiation potential and immunophenotypic markers. The sEV were isolated by ultracentrifugation and characterized by nanoparticle tracking analysis and western blot. Its miRNA cargo was examined by quantitative PCR analysis for miR-26a, 126a, and 296. Wistar rats were submitted to UUO procedure and concomitantly treated with sEV secreted by BM-MSC from the untrained andtrained rats. The kidney tissue from all groups was evaluated for fibrosis mediators (transforming growth factor beta1 and collagen), CD34-angiogenesis marker, and hypoxia-inducible factor 1 alpha (HIF-1α).

**Results:**

Treadmill training stimulated in BM-MSC the production of sEV loaded with pro-angiogenic miR-296. The treatment with this sEVin UUO-rats was able to attenuate collagen accumulation and increase CD34 and HIF-1α in the kidney tissue when compared to untrained ones. Tubular proximal cells under hypoxia and exposed to BM-MSC sEV demonstrate accumulation in HIF-1α and NFR-2 (nuclear factor erythroid 2-related factor 2), possibly to mediate the response to hypoxia and oxidative stress, under these conditions.

**Conclusion:**

The BM-MSC sEV from trained animals presented an increased nephroprotective potential compared to untrained vesicles by carrying 296-angiomiR and contributing to angiogenesis in UUO model.

## Background

Chronic kidney disease (CKD) results in a gradual loss of kidney function, leading to end-stage kidney disease, in which the only treatments available are dialysis or kidney transplantation [1]. Structural loss of renal function can result from glomerulosclerosis, tubular interstitial fibrosis, or both [2,3]. Glomerular injury and tubulointerstitial injury are associated with the recruitment of inflammatory cells and profibrotic cytokines. Although sclerotic and fibrotic lesions lead to kidney damage, the reduction in glomerular filtration rate correlates with tubular interstitial fibrosis [2-5]. Renal fibrosis is characterized by the activation and proliferation of renal interstitial fibroblasts and the deposition of extracellular matrix components [5]. Mesenchymal stromal cell (MSC) therapy is the most advanced cellular therapy to date. The efficacy of MSC-based cell therapy has been demonstrated for a broad spectrum of indications *in vivo* and *in vitro*, including kidney diseases [6-8].

According to the International Society for Cell & Gene Therapy (ISCT®), MSC must have three characteristics for their characterization. First, they must express CD90, CD73, and CD105 surface markers and the absence of CD45, CD34, CD11b, CD19, and HLA-DR markers. Second, they must be able to adhere to a plastic surface under culture conditions, and third, they must be able to differentiate into chondroblasts, osteoblasts, and adipocytes *in vitro* [9-11].

Mesenchymal stromal cells can be conditioned through specific minerals and vitamins to increase protective capacity. This strategy allows the cell to have improved insertion properties and survive in hostile microenvironments [12,13]. In addition, studies have shown that MSC may be conditioned by hypoxia. These effects on MSC efficiency and proliferation are still controversial and may depend on seeding density, oxygen concentration, culture conditions, and the sources of MSC [14,15].

Nevertheless, the donor characteristics are not considered. Most studies extract BM-MSC from adult and healthy animals, but no study evaluated the effects of physical exercise on BM-MSC, especially in extracellular vesicles (EVs). In addition, the benefits of physical activity in CKD are well known [16,17]. It has been reported that physical activity is an essential intervention for patients on hemodialysis to improve their physical performance [18].

Many studies attempt to characterize the paracrine communication mediated by BM-MSC, and extracellular vesicles play a central role [19,20]. One of the most significant advances of recent research showed vesicle’s ability to transport and transfer ribonucleic acid (RNA) and microRNA (miRNA) to recipient cells, thus modulating their protein expression pattern [19, 21-23].

The term extracellular vesicles (EVs) includes exosomes (30-100 nm), microvesicles (100-1000 nm), and apoptotic bodies (50-5000 nm), which are differentiated based upon their biogenesis, size, release pathways, function, and content [24-27]. Researchers use the term “small extracellular vesicles”, since the isolation of a pure population of exosome through the method used in the present study is difficult. Therefore, we will use the term“small extracellular vesicles” (sEV) to refer to EVs less than 30-150 nm in diameter, according to the updated guidelines of the International Society for Extracellular Vesicles of 2018 (MISEV2018) [27].

EVs can transport proteins, lipids, RNA, mainly miRNA, and mediate intercellular communication [28,29]. EVs express evolutionarily conserved proteins, including tetraspanins (CD9, CD63, CD81), Alix, TSG101, and HSP70 [27], as well as specific proteins that appear to reflect their original cell [30,31]. 

BM-MSC can also release extracellular vesicles after stimulation, being able to exert their therapeutic effects in a paracrine/endocrine manner [19,32,33]. Several studies have shown that EVs derived from MSC, mainly from bone marrow, can promote tissue repair/recovery and reduce inflammation in different kidney injury models [34-40]. So, it is reasonable to suggest that physical exercise can improve the protected effect of EVs secreted by bone marrow mesenchymal stromal cells.

UUO is an experimental model widely used to simulate kidney disease with tubulointerstitial fibrosis, chronic inflammation caused by continuous ureteral obstruction, leading to progressive renal function loss [41-44], considered then an aggressive experimental model of chronic kidney disease. Therefore, this work aims to evaluate the treatment with sEV secreted by BM-MSC from trained rats in their protective capacity in chronic kidney disease induced by unilateral ureteral obstruction (UUO).

## Methods

### Animals and experimental design

Male Wistar rats weighing between 190 and 210 grams at 30 days of age were used. The animals obtained were housed in controlled temperature (25ºC/77 ºF) room and had free access to water and rat chow (Nuvilab, Brazil). The study was approved by the Ethical Committee of Experimental Animals of Universidade Federal de São Paulo (CEEA - protocol. nº 8628/0000050814). The protocol approved was in agreement with the Brazilian guidelines for scientific animal care and use. 

After acclimation for seven days, the rats were randomly assigned into two groups (n=3 each). The control group not submitted to physical activity was called the untrained group (UT), and the exercise training group was called trained group (T). 

The functional capacity was measured in the trained group using the maximal exercise test, as described in the literature [45,46]. The rats were submitted to an adaptation period on the treadmill, at a rolling speed of 5 meters for 10 minutes (m/min), with 5 m/min increments every 3 minutes. 

The criterion for exhaustion in the animals was an inability to cope with the speed of the treadmill. Thus, we opted for the maximal running test and exercise training for an intensity of 65% to 70% of the maximum speed reached in the test to characterize high-intensity exercise. 

Finally, each training session was divided into five minutes of warming up and 40 minutes of exercise. There were 5 sessions per week for 4 months.

The untrained and trained rats were euthanized 121 day safter the beginning of the experimental protocol through an intraperitoneal injection of a toxic dose of 10 mg/kg of xylazine (Agribrands do Brasil, Brazil) and 90 mg/kg of ketamine (Agribrands do Brasil, Brazil).

### Culture and characterization of bone marrow mesenchymal stromal cells (BM-MSC)

The bone marrow (BM) was obtained fromthe untrained group (UT) and ofthe trained group (T). Briefly, rats were sacrificed, and femurs and tibias were aseptically removed. The BM was flushed from the shaft of the bone with DMEM medium (Sigma, USA) containing 5% fetal bovine serum (FBS) (Invitrogen, Scotland) plus penicillin/streptomycin (100 U/mL to 0.1 mg/mL; Invitrogen). BM-MSC were recovered by centrifugation (1500g for 30 minutes) in a density gradient with Ficoll Hystopaque (Sigma, USA), followed by their tendency to adhere tightly to plastic culture dishes, as previously described [8]. BM-MSC were plated in DMEM plus 20% FBS and penicillin-streptomycin (100 U/mL to 0.1 mg/mL), allowed to adhere for 24 hours (h) and maintained in regular cultures conditions of once-a-week culture medium change and twice a week trypsinization until were used between 4-6^th^ passages for the characterization protocol.

The differentiation potential of BM-MSC into osteocytes and adipocytes was performed through of Mesencult osteogenic and adipogenic differentiation kit (Stemcell, Canada), according to manufacture instructions. After the incubation period, the cells were fixed with 10% formalin for 20 min at room temperature, and mineralization (presence of calcium-rich hydroxyapatite) of the extracellular matrix was assessed by staining for 20 min with 5% wt/vol Von Kossa Silver, adjusted to pH 4.1 with ammonium hydroxide (all reagents were from Sigma) [8, 47,48]. Adipogenic differentiation was visualized in phase-contrast microscopy by the presence of highly refractive intracellular lipid vacuoles. Oil Red O (Sigma, USA) staining was used to assay the accumulation of lipid droplets in these vacuoles [8,49].The results were shown through photomicrographs using Nikon fluorescence microscopy (Nikon, Japan).

The BM-MSC markers, anti-CD90-FITC, and anti-CD45-PE (all from BD Pharmagen, USA) were analyzed by flow cytometric analysis, and one negative control tube with cell suspension was also used as control. The cells were incubated with purified antibody, washed twice with phosphate-buffered saline (PBS) buffer, and incubated with anti-rabbit antibody conjugated to Alexa Fluor 488 (from Becton Dickinson Company, USA) for 20 minutes. After incubation, were rewashed with PBS buffer and resuspended in 500 µl PBS for the FACS analysis. The results from all tubes were analyzed, and the results are expressed as percentages.

### Small extracellular vesicles secreted by BM-MSC and their characterization

This method is in according with “Comparison of Minimal Information for Studies of Extracellular Vesicles 2018” (MISEV2018) [27].The small extracellular vesicles (sEv) were isolated from BM-MCS according to the following protocol.

The conditioned media (CM) was obtained from untrained group (UT) and trained group (T). BM-MSC was maintained under culture conditions and serum-free culture medium for 24 h. Then, the CM was collected and immediately subjected to centrifugation at 800 g for 5 min and 2000 g for 10 min, and the supernatant was filtered through a 0.22µm pore filter. Subsequently, the filtered medium was subjected to two ultracentrifugations at 100.000 g 4ºC for 1h, the last one in PBS. After discarding the supernatant, the pellet was collected and used or maintained at -80 ^o^C.

The small extracellular vesicles (sEV) secreted by BM-MSC from the untrained group (UT) is called sEV-UT, while small extracellular vesicles (sEV) secreted by BM-MSC from the trained group (T) is called sEV-T. The parameter for small extracellular vesicles dose determination was protein concentration, assessed by Lorry-method and showed previously [50]. Thus, the protein dosage infers indirectly in the amount of sEV.

The CD63, amarker of small extracellular vesicles [27],was analyzed by western blotting. The samples of sEV-UT and sEV-T groups were separated by 10% SDS-PAGE and transferred to nitrocellulose membranes using a Trans-Blot® Turbo (Bio-Rad, USA). Nonspecific binding sites were blocked with 5% albumin (v/v) in a TBS buffer. The immunoblots were incubated overnight at 4 °C with rabbit CD63 antibody (1:300, Santa Cruz Biotechnology, USA). After washing three times with TBS-T, the membranes were incubated for 2h at 4 °C in anti-rabbit HRP-conjugated secondary antibodies (1:100.000; Cell Signaling, USA). Immunoreactive protein bands were visualized using Clarity™ Western ECL Substrate detecting kit (Bio-Rad). Images were obtained and analyzed by Chemiluminescence with Amersham Imager 680 (GE, USA).

The samples of sEV-UT and sEV-T groups were resuspended in PBS and inserted through a syringe coupled to the Malvern NanoSight NS300 (NanoSight, UK) for analysis. The capture settings were camera Level: 13 (NTA 3.0 Levels), number of frames: 374, temperature: 23.2 - 23.3 ^o^C, and the analysis settings were 5 threshold level. The device measured the concentration of the particles/mL through three videos of 10 seconds each one. The particles movement provides size and distribution according to its Brownian movement. The results are expressed as particles/mL.

The miR-26a-5p, miR-126a-5p and miR-296-3p were analyzed using the TaqMan™ Advanced miRNA Assaykit (Applied Biosystems, CA). The miRNA expression levels were analyzed by qPCR: 200 ng of RNA was reverse-transcribed (TaqMan™ MicroRNA Reverse Transcription Kit), and the complementary DNA was used to detect and quantify specific miRNAs within small extracellular vesicles by qRT-PCR using TaqMan™ Advanced miRNA Assay kit (Applied Biosystems, CA), according to manufacture instructions. The results are expressed as arbitrary units (AU).

### Unilateral ureteral obstruction (UUO)

Eighteen male Wistar rats weighing between 190 and 210 grams at 30 days of age were anesthetized for the UUO-procedure. A low midline abdominal incision was made, the ureter was mobilized and isolated with minimal dissection and connected with two 6.0 silk threads (Bioline, USA) at the ureterovesical junction. Six animals submitted to this procedure were called unilateral ureteral obstruction (UUO) group. Six rats submitted to UUO were concomitantly treated with the small extracellular vesicles (sEV) secreted by BM-MSC and called untrained group (UUO-sEV-UT). Additionally, six rats submitted to UUO were concomitantly treated with sEV secreted by BM-MSC from the trained group (T), and this group was called UUO-sEV-T. Two rats underwent an identical surgical procedure, without ureteral ligation and with an injection of only PBS solution, and were called the Sham group (data not shown).

The small extracellular vesicle treatment was concomitant with the UUO-procedure. The renal artery was carefully dissected, and rats received 1×10^6^small extracellular vesicles resuspended in PBS that were injected into renal artery.On day 7 post-surgery (UUO-procedure), the animals were euthanized by intraperitoneal injection of a toxic dose of 10 mg/kg of xylazine (Agribrands do Brasil, Brazil) and 90 mg/kg of ketamine (Agribrands do Brasil, Brazil).

### Immunohistochemistry

Kidneys were dissected along the non-hilar axis, fixed in 10% phosphate-buffered formalin (Erviegas, Brazil). Afterward, kidney sections were fixed with 4% buffered paraformaldehyde and embedded in paraffin (Erviegas, Brazil). Next, 4 μm thick sections were prepared in Cryostat, and kidney slices were deparaffinized and rehydrated. Endogenous peroxidase activity was blocked with 5% hydrogen peroxide/absolute methanol for 10 min. To expose the antigens, kidney sections were boiled in a target retrieval solution [1 mmol/L tris(hydroxymethyl)aminomethane] pH 9.0, with 0.5 mM ethylene glycol tetra acetic acid for 10 min. Nonspecific binding was prevented by incubating with phosphate-buffered saline (PBS) containing 1% FBS albumin, 0.05% saponin, and 0.2% gelatin. Kidney sections were incubated with primary antibodies against transforming growth factor beta1 (TGF-β1: fibrosis mediator), hypoxia-inducible factor 1 alpha (HIF-1α), and CD34-angiogenesis marker (1:200, rabbit anti-rat; ABCAM, USA). Protein expression was determined using a streptavidin peroxidase kit (Dako, USA). Sections were stained with diaminobenzidine for antibody detection and then counterstained with hematoxylin. Spots were absent in negative control sections. Digital photomicrographs were taken with a Leica DM 1000 upright microscope connected to a workstation computer through a Leica DFC 310 FX, LAS 3.8 Microscope Camera (Leica, Switzerland). Ten photomicrographs were taken along the kidney cortex, and the light brown staining was quantified (LAS software, version 3.8) and averaged for each rat. The values obtained are expressed as percentage/stained area.

Fibrillar collagen accumulation (fibrosis) by collagen-specific Picrosirius Red staining as evaluated as previously reported [51,52]. Collagen volume fraction in the kidney was quantitated as previously reported and calculated as the percentage of connective tissue (red) areas compared to the sum of connective tissue area and non-connective tissue in all fields of the kidney section (20 sections/kidney). The values obtained are expressed as percentage/stained area.

### Cell culture and hypoxia

Rat proximal tubular cells (RPTEC) were grown in RPMI-1640 medium (Sigma, USA) supplemented with 10% FBS (Gibco, USA), 24 mM of sodium bicarbonate, 10 mM of N’-2-hydroxyethylpiperazine- N’-2-ethanesulfonic acid, and 10.000 U/L of penicillin/streptomycin. 

Semi confluence cell cultures (norm group) were maintained in normoxic conditions of at 37°C, 5% carbon dioxide, 21% oxygen_,_and 74% nitrogenand humidified atmosphere during 48h, while cells subjected to hypoxia (hyp) were maintained in a Hypoxia Incubator Chamber (Stem Cell Technologies, Canada) in the presence of a gas mixture of 1% oxygen, 5% carbon dioxide, and 94% nitrogen_,_ for 48h. Cells under hypoxic conditions were subsequently divided into two more groups, respectively: RPTEC under hypoxic conditions and treated with 50µg/mL of small extracellular vesicles (sEV) secreted by BM-MSC from the untrained group (Hyp+sEV-UTgroup) and RPTEC under hypoxic conditions and treated with small extracellular vesicles (sEV) secreted by BM-MSC from the trained group (T): Hyp+sEV-T group.

Hypoxia conditions were verified by immunofluorescence, using a method described by Convento et al. [53] for HIF-1α. The antioxidant enzyme nuclear factor erythroid 2-related factor 2 (Nrf2) also was verified by immunofluorescence [53]. The microscope images obtained were calculated using the Leica DFC 310 FX image analysis software (Leica, Switzerland) and are expressed as percentages/stained area.

### Statistical analysis

The descriptive statistical analysis of the data was performed using the Action Stat software (version 3.3.2) for Windows. The results were expressed as mean ± SE. Data were analyzed by one-way analysis of variance (ANOVA) followed by the Tukey test or Student t-test, and p < 0.05 was considered statistically significant. 

## Results

### Evaluation of maximal exercise test

The untrained group (UT group) showed a maximal exercise test (MET) of 19.2 ± 0.7 m/min, while the animals in the trained group (T group) showed a MET of 35. 4 ± 0.6 m/min, an improvement of 54% when comparedto the UT group (Table 1).

**Table 1.** Maximum exercise test.

MET	Trained group (T)	Untrained group (UT)	P-value
	35.40 ± 0.60	19.20 ± 0.74	p < 0.1

### Characterization of BM-MSC

**Figure 1.**
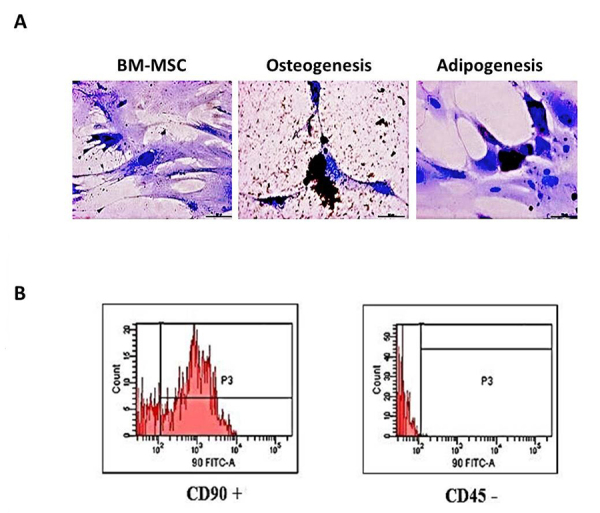
Characterization of bone marrow mesenchymal stromal cell (BM-MSC). **(A)** Photomicrograph of cell differentiation of BM-MSC cells into adipocytes and osteocytes. **(B)** Flow cytometry for CD90 and CD45 of BM-MSC cells.

BM-MSC was obtained from trained and untrained rats. These cells in culture, after 21 days of induction, confirmed the potential differentiation into adipocytes and osteocytes (Figure 1A). Also, flow cytometry analysis showed a positive marker for the CD90 and a negative marker for CD45 (Figure 1B), as expected.

### Characterization of small extracellular vesicles

**Figure 2.**
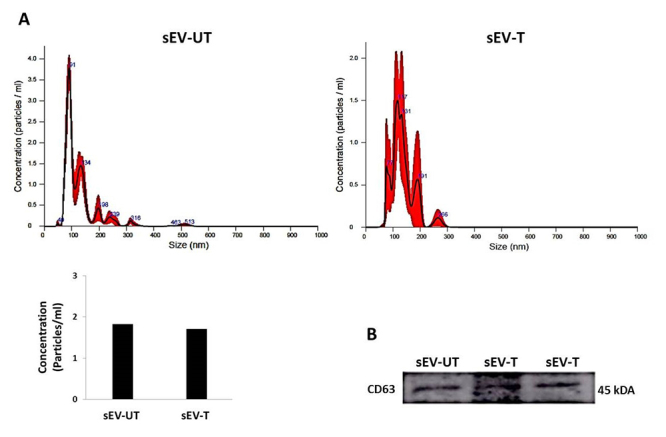
Characterization of small extracellular vesicles (sEV). **(A)** Nanoparticle tracking analysis (NTA) distribution and concentration (particles/mL) of sEV secreted by bone marrow mesenchymal stromal cell (BM-MSC) from the untrained rats (sEV-UT) and trained rats (sEV-T). **(B)** Photomicrograph of the western blot analysis for CD63 of sEV secreted by BM-MSC from the untrained rats (sEV-UT) and trained rats (sEV-T).

The small extracellular vesicles size was analyzed via nanoparticle tracking analyses (NTA).The sEV-T and sEV-UT groups showed, respectively, a mode of 97 nm and 116 nm, which correspond to the size range for small extracellular vesicles. Interestingly, there was no significant difference in the size of small extracellular vesicles secreted by BM-MSC from trained (T group) or untrained (UT group) rats. Additionally, there was no difference in the concentration of the nanoparticles between sEV-T (1.71^11^ particles/mL) and sEV-UT (1.82^11^ particles/mL) groups (Figure 2A).The CD63 small extracellular vesicles marker was analyzed by western blotting and expressed in both groups (Figure 2B).

### *In vivo*: rats submitted to unilateral ureteral obstruction (UUO) and the effects of BM-MSC small extracellular vesicle treatment

**Figure 3.**
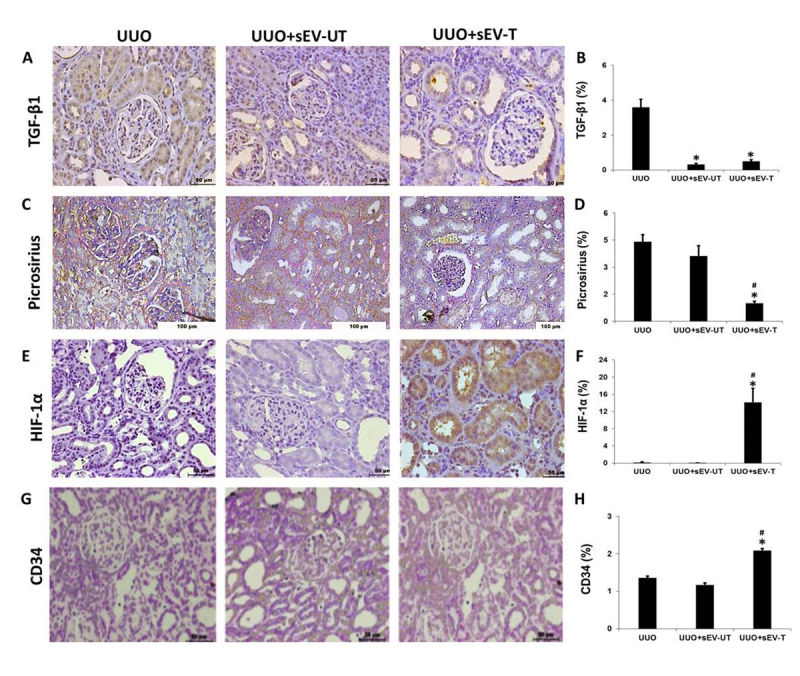
Immunohistochemistry images and quantitative analyses, respectively, for **(A,B)** TGF-β1, **(C,D)** Picrosirius red, **(E,F)** HIF-1α, and **(G,H)** CD34. The significance level for a null hypothesis was set at 5% (p < 0.05). *All groups compared to the UUO group, ^#^UUO+sEV-T compared to the UUO+sEV-UT.

As shown in Figure 3A and 3B, in the renal cortex of the left kidney, after 7 days of UUO, there was TGF-β1 accumulation (3.76 ± 0.38%). The administration of small extracellular vesicle (sEV) prevents its expression in both treated groups (UUO+sEV-T: 1.34±0.38%, and UUO+sEV-UT: 0.82 ± 0.19%) (p<0.05) when compared with the UUO. 

Using Picrosirius Red staining technique, we observed that the percentage of collagen-stained area in the UUO+sEV-T group (4.2 ± 0.4%) showed a significant decrease (p< 0.05) in comparison to the UUO (7.8± 0.6%) and UUO+sEV-UT groups (7.4± 0.5%), in Figure 3CC and 3D.

The analysis of the hypoxia marker HIF-1α showed significant increase in the percentage of stained area (p< 0.05) in UUO+sEV-T group (14.16 ± 10.68 %) in comparison to the UUO (0.16 ± 0.46 %) and UUO+sEV-UT groups (0.04 ± 0.5%), as presented in Figures 3E and 3F.

Also, the endothelial cell marker CD34 presented a significant increase in percentage of stained area (p< 0.05) in the kidney of UUO+sEV-T group (2.09 ± 0.18 %) when compared to the UUO (1.36 ± 0.16%) and UUO+sEV-UT groups (1617 ± 0.17 %), as shown in Figures 3G and 3H.

### *In vitro*: the response of proximal tubule epithelial cell (RPTEC) to hypoxia and the effects of small extracellular vesicle treatment

**Figure 4.**
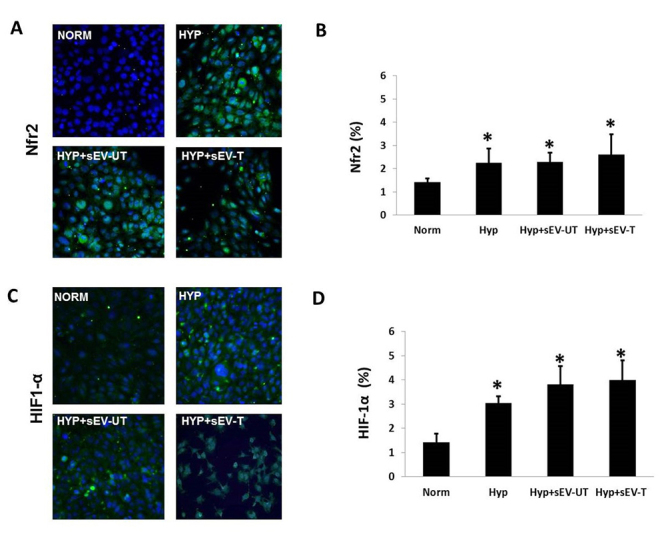
Immunofluorescence images (FITC refers to green fluorescence whereas blue indicates nuclei), and quantitative analyses, respectively, for **(A,B)** Nrf2 and **(C,D)** HIF-1α. The significance level for a null hypothesis was set at 5% (p < 0.05). *All groups compared to the Norm group.

It is difficult to reproduce *in vitro* the conditions observed in UUO model, however, an important component of this model is hypoxia, observed mainly in the proximal tubular cell. Thus, in order to better evaluate the effects of sEV on the proximal tubule cell, we used the cell culture model submitted to normoxic and hypoxic conditions and treated with sEV. Figure 4 is shown the Nrf2 (A,B) and HIF-1α (C,D) protein synthesis (immunofluorescence assay) with their respective graphical quantification. The results showed an increase in HIF-1α, and Nrf2, in all experimental groups under hypoxic conditions when compared to the group maintained in normoxia. Nevertheless, between experimental groups under hypoxic conditions, there was a significant difference in HIF-1α and Nrf2.

Although we did not observe a difference between the groups submitted to hypoxia and treated with sVE, this result may suggest that the concomitant increase in HIF-1α and Nrf2 in the proximal tubule cell may mediate a response to hypoxia and associated oxidative stress through antioxidant enzymes stimulation [54].

### *In vitro*: evaluation of miRNAs in BM-MSC cell-derived small extracellular vesicles secreted by BM-MSC from trained rats (sEV-T) and untrained rats (sEV-UT)

**Figure 5.**
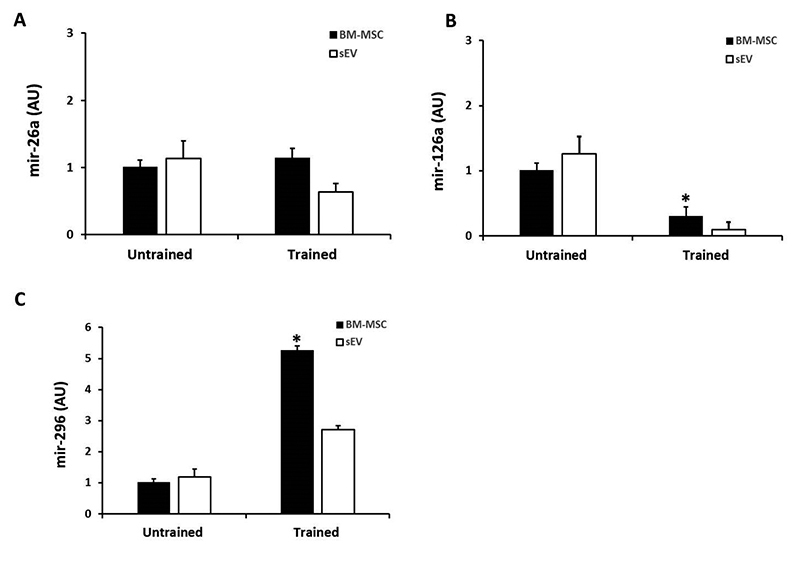
Quantitative polymerase chain reaction (qPCR) showing **(A)** miR-26a, **(B)** miR-126a, and **(C)** miR-296. The significance level for a null hypothesis was set at 5% (p < 0.05). *BM-MSC of trained rats compared to the BM-MSC of untrained rats.

When the microRNA was evaluated in sEV, the qPCR analysis showed miR-26a, miR-126a, and miR-296-expression (Figure 5) in all experimental groups.

In relation to miR-26a (Figure 5A), there was no significant difference associated with the small extracellular vesicles and BM-MSC from trained rats (sEV-T) and untrained rats (sEV-UT).

Nonetheless, there was a decrease in miR-126a (Figure 5B) expression in the BM-MSC cells and in the small extracellular vesicles secreted by BM-MSC from trained rats (sEV-T) when compared to the BM-MSC cells and in the small extracellular vesicles secreted by BM-MSC from untrained rats (sEV-UT) 

Interestingly, when we compared miR-296 expression in sEV, this angiomir was significantly increased in BM-MSC cells obtained from trained rats and in its small extracellular (sEV-T) in comparison to cells and sEV from untrained rats (sEV-UT), suggesting that physical activity can increase the expression of this angiomiR (Figure 5C).

## Discussion

UUO is an experimental model widely used to simulate kidney disease with tubulointerstitial fibrosis, chronic inflammation caused by continuous ureteral obstruction, leading to progressive renal function loss [41-44], considered then an aggressive experimental model of chronic kidney disease. 

Several studies underscored the antifibrotic and renoprotective properties of BM-MSC-derived sEV in UUO model and showed a potent reduction of TGF-β1 signaling [55-57]. TGF-β1 can induce fibrotic genes and apoptosis in tubular and glomerular cells, acquiring a myofibroblastic phenotype essential for the formation of fibrosis [53,58,59]. In our study, the treatment with sEV secreted by murine BM-MSC, independent of physical activity, showed a decrease in the levels of TGF-β1 in the rats submitted to UUO, suggesting that sEV-treatment can contribute to the reduction of renal fibrosis via TGF-β1. 

UUO causes the development of profibrotic features, also reflected by increased collagen deposition. In this study, the use of small extracellular vesicles secreted by BM-MSC from trained rats decreased collagen deposition in kidney tissue of rats subjected to UUO.

The CD34 is the most described marker in circulating angiogenic cells that are known for their progenitor cell properties. In our study, we observed an increase in CD34 expression in kidney tissue, only in UUO-rats that received small extracellular vesicles secreted by BM-MSC from trained rats. Sahoo et al. [60] demonstrated that human CD34^+^ stromal cells are capable of secreting sEV with angiogenic activity in isolated endothelial cells and in vessel growth murine models. Also, Landers-Ramos et al. [61] demonstrated a differential paracrine effect in circulating CD34^+^ angiogenic cells associated with frequent physical activity, corroborating our findings.

Additionally, Masum et al. [62] demonstrated that mice with UUO presented decreased CD34^+^ cells in tubulointerstitial area. They conclude that the injury and/or loss of capillaries in the tubular areas contribute to the progression of tubule interstitial injury, fibrosis, and tubular damage, especially in kidneys with UUO [62]. Taking it into account, our results suggest the potential of the sEV secreted by BM-MSC from trained rats when compared with untrained animals.

HIF-1α is also known to play an important role in the transcription of angiogenic genes [63-66]. Several evidences have already shown that HIF-1α is a central regulator of renal fibrosis in different pathological conditions [67-70]. However, it remains a controversy whether HIF-1α promotes or antagonizes renal fibrosis. In fact, there is an important regulatory role for HIF-1α in renal fibrogenesis, and if it has pro or antifibrotic effects will depend on which, where, and when it is activated [70]. 

Kapitsinou et al. [68] showed that HIF-1α accumulation through pharmacological prolyl hydroxylase inhibitors immediately before ischemia can inhibit fibrosis. However, this effect was not observed when the inhibition was made post-ischemia. Also, there was a decrease in fibrosis and an improvement in the tubulointerstitial lesion when HIF-1α was activated pharmacologically in a remaining kidney model [70-72].

Kobayashi et al. [73] showed that the genetic activation of HIF-1α suppresses fibrogenesis in a UUO model. In our study, we observed an increase in HIF-1α expression in kidney tissue, only in UUO-rats treated with small extracellular vesicles secreted by BM-MSC from trained rats.

The sEV secreted by bone marrow mesenchymal stromal cells carry microRNAs and deliver them into kidney. These miRNAs induce target mRNA degradation or inhibit target mRNA translation. This process can mediate antifibrotic, anti-inflammatory, and anti-apoptotic actions in UUO and in ischemic injury [19]. To prove this hypothesis, we evaluated three nephroprotective miRNAs in sEV secreted by bone marrow mesenchymal stromal cells.

Cantaluppi et al. [74] showed that sEV released from CD34-positive endothelial progenitor cells (EPCs) contributes to angiogenesis and protects the kidney from ischemia-reperfusion injury by miR-296-dependent reprogramming of resident renal cells. Our work showed a significant increase in the expression of miR-296 only in the sEV secreted by BM-MSC from trained rats. Thus, it is reasonable to suggest that the transfer of BM-MSC sEV from trained animals can have an improved protective effect in comparison to untrained animals, improve the also be an alternative to stimulus the kidney's angiogenesis. 

Studies suggest that miR-26a expression was much lower in humans with renal vascular disease comparing healthy ones [75]. The decrease in miR-26a expression in kidney tubular cells elevates the possibility of cell apoptosis, but this process was reversed by co-culture with adipose tissue-derived mesenchymal stem cells [76]. Liang et al. [77] showed that ischemia-reperfusion injury increased macrophages and neutrophils infiltration into renal tissues to cause tubular injury. Interestingly, the expression level of miR-26a was also lower in ischemia-reperfusion injury tissues than in normal tissues. Our results showed miR-26a-expression in sEV secreted by BM-MSC from trained and untrained rats was decreased. This finding suggest that miR-26a is not involved in sEV effects in our model. 

The miR-126a is a pro-angiogenic miRNA known to be involved in the modulation of angiogenesis and vascular integrity [78,79]. It has already been shown that physical activity can increase the expression of this miRNA in cardiac [80] and vascular tissues or swimming and treadmill trained rats, respectively [81]. Another study by the same group concluded that sEV derived from mouse progenitor endothelial cells submitted to moderate physical activity has beneficial effects that correlate with sEV transported by miR-126a [79]. However, our results did not show an increase in miR-26a-expression in sEV secreted by BM-MSC from trained when compared with untrained rats. 

Our results support the hypothesis that sEV secreted by bone marrow mesenchymal stromal cells from trained rats stimulates an increase in the pro-angiogenic miR-296. In the kidney tissue of UUO-rats that received this sEV, there was an increase in CD34, which is the most described marker in circulating angiogenic cells [60]. The HIF-1α, which is also known to play an important role in transcriptions of angiogenic genes [63-65], was increased in the kidney tissue of UUO-rats that received this sEV. The small extracellular vesicles obtained by bone marrow of trained animals can possibly contribute to angiogenesis, which in turn promotes revascularization in the kidney.

Together, our results suggest that sEV obtained from BM-MSC of trained rats have improved protective effects when compared to vesicles obtained from cells of untrained animals, suggesting that characteristics of the donor can possibly determinate the success of the BM-MSC transplant or its sEV treatment. Nevertheless, new studies are needed to clarify the mechanisms involved in sEV obtained from trained animals, but also other studies are needed to evaluate necessary the effect of diabetes, hypertension, and diet in the nephroprotective effect of stromal cell sEV.

## Conclusion

In conclusion, the treatment with the small extracellular vesicles secreted by bone marrow mesenchymal stromal cells from trained rats carries pro-angiogenic miR-296 and delivers them into rats subjected to unilateral ureteral obstruction, possibly corroborating angiogenesis, via CD34 and HIF-1α.

### Abbreviations

BM: bone marrow; BM-MSC: bone marrow mesenchymal stromal cells; CEEA: committee of experimental ethical animals; CKD: chronic kidney disease; CM: conditioned media; EVs: extracellular vesicles; FBS: fetal bovine serum; H: hour; HIF-1α: hypoxia-inducible factor 1 alpha; Hyp: hypoxia; MET: maximal exercise test; min: minutes; miRNA: microRNA; Nfr2: nuclear factor erythroid 2-related factor 2; Norm: normoxia; NTA: nanoparticle tracking analysis; PBS: phosphate-buffered saline; qPCR: quantitative polymerase chain reaction; RNA: ribonucleic acid; RPTEC: rat proximal tubular cells; sEV: small extracellular vesicles; T: trained; TGF-β1: transforming growth factor beta1; UT: untrained; UUO: unilateral ureteral obstruction. 
